# Importance of Life-Long Alerts in Electronic Medical Report Systems for Patients With Positive Antigen Screening

**DOI:** 10.7759/cureus.22561

**Published:** 2022-02-24

**Authors:** Bianca C Woodruff, Lazar Popilevsky, Roni Mendonca, Michael Girshin

**Affiliations:** 1 Department of Anesthesiology, Metropolitan Hospital Center, New York, USA

**Keywords:** perioperative transfusion, perioperative anesthesia, emergent intraoperative hemorrhage, transfusion reaction, alloimmunization

## Abstract

Perioperative red blood cell transfusions are common. Antigen screening, performed via indirect Coombs test, is required prior to red blood cell transfusion. False-negative test results can lead to acute or delayed hemolytic transfusion reactions. This case report emphasizes the importance of using life-long alerts in a hospital’s electronic medical record system for patients with positive antigen screening to prevent false-negative preoperative antigen results from leading to hemolytic transfusion reactions.

## Introduction

With an estimated incidence of 6.2%, perioperative red blood cell (RBC) transfusions are common [1]. Although many patients undergoing lung lobectomy may require one or more perioperative RBC transfusions, a multicenter study conducted by the College of American Pathologists surprisingly found that 9% of type and screen tests were not completed before the start of surgery [2]. If compatible units are unavailable on short notice, an unexpected, positive, perioperative alloimmunization test result can challenge the continuation of ongoing surgery [2]. In such cases, uncross-matched type O negative blood may be given to patients with positive antigen screening after life-threatening hemorrhage [3], but the benefit-risk ratio of acute hemorrhage versus acute or delayed hemolytic transfusion reactions must be carefully assessed. Life-long alerts in a hospital’s electronic medical record (EMR) system should be issued for patients with positive antigen screening history to help protect patients and prevent surgery discontinuation or delays.

## Case presentation

A 56-year-old female seen in a preanesthesia clinic with American Society of Anesthesiologists (ASA) physical status class 3 presented for an elective right upper lung lobectomy via thoracotomy approach and mediastinal lymph node dissection. Her past medical history was remarkable for controlled hypertension, type 2 insulin-dependent diabetes mellitus, chronic obstructive pulmonary disease (COPD), class 2 obesity (body mass index of 36), former tobacco use for 30 years, controlled schizophrenia, gravida 11, para 11, and squamous cell carcinoma of the right upper pulmonary lobe. No previous RBC transfusions had been received. The preoperative cardiac evaluation showed normal sinus rhythm and normal left ventricular ejection fraction. Pulmonary function tests confirmed COPD with persistent airflow limitations. Preoperative laboratory tests were unremarkable with a hemoglobin level of 11.9 g/dl and a hematocrit level of 36.9%. Preanesthetic physical exams, including dental, cardiovascular, pulmonary, neurological, and abdominal status, were consistent with the past medical history. The Mallampati score was 2. A preoperative type and screen test, ordered during preoperative evaluation by the anesthesiology provider and performed one week prior to surgery, revealed blood type A positive with negative antibody screen.

On the day of surgery, a second type and screen for crossmatching was done. General anesthesia was provided with 2 mg midazolam, 20 mg etomidate, 50 mg propofol, 50 mcg fentanyl, 40 mg lidocaine, and 50 mg rocuronium. The patient was intubated with a left-sided double-lumen tube and surgery was started without complication. After the surgery team released pulmonary adhesions, continuous profuse bloody oozing occurred with an estimated blood loss of 300 ml. After discussion with the surgical team, the decision was made to request packed RBCs from the blood bank in case further significant blood loss occurred. The blood bank informed the anesthesiologist that the antibody screen was positive with anti-U and that only one unit of cross-matched blood was immediately available. Since dissection and division of the branches of the right superior pulmonary artery and right superior pulmonary vein had already been performed without bleeding complication, the decision was made to continue surgery. Division of the right upper lobe bronchus was uneventful. Hemodynamically stable throughout the rest of the procedure, and without further significant blood loss, the patient did not require a blood transfusion. After switching the double-lumen tube to a single-lumen tube, the intubated and analgosedated patient was brought to the surgical intensive care unit.

A close review of the patient’s past medical records from other hospitals, which were not available during the preoperative evaluation, revealed a positive antibody screen two years prior showing anti-U.

## Discussion

Patients undergoing any elective surgery may suddenly require an RBC transfusion. Thoracotomy with lobectomy carries a significant risk of perioperative hemorrhage. The Association for the Advancement of Blood and Biotherapies (AABB) guidelines recommend that type and screen testing is only valid for up to three days [4]. Finding compatible units for transfusion in the setting of rare alloimmunization may require extended time, up to days, for additional crossmatching. A multicenter study by the College of American Pathologists showed that 25% of type and screens were drawn on the day of surgery and 9% were not completed before surgery started. Ideally, the antibody screen should be completed prior to the beginning of the operation. Positive antibody screens occurred in 2% of type and screens. Of positive antibody screens, 5.7% led to discontinuation or delay of surgery [2].

An indirect Coombs test, also called indirect antiglobulin test, antibody screening, or crossmatching, is used to detect antibodies against foreign RBCs in a patient’s serum or plasma prior to a blood transfusion. The indirect Coombs test is a two-stage test. During the first stage, the patient’s serum is incubated with a wide range of washed foreign RBCs of known antigenicity to include a full range of surface antigens. In the second stage, the RBCs are washed three or four times with isotonic saline solution and afterward are incubated with an anti-human globulin, which is known as the Coombs reagent to distinguish the presence or absence of immunoglobulins on the surface of the RBCs. If antibodies have bound to RBC surface antigens in the first stage, the RBCs will agglutinate with the Coombs reagent in the second stage. The indirect Coombs test will then be declared positive [5].

Reasons for false-negative results of the indirect Coombs test are numerous. These include an insufficient quantity of antibodies on RBCs, improper incubation temperature (including during transport to external laboratories), under-centrifugation, delay in adding Coombs reagent or adding expired Coombs reagent, incorrect quantity of Coombs reagent, improper antigen to antibody ratio, and elution of low-avidity antibodies from RBCs during washing [6]. Buchta et al. showed that antibody screening produces 0.16% false-negative results [7].

Importantly, once having a single positive antibody screen, the patient should be considered positive, even if later type and screen testing comes back negative. In our case, the patient’s past medical records from other hospitals with a positive antibody screen for anti-U were not available during the preoperative evaluation. We relied entirely on our own type and screen that was negative prior to the procedure, but apparently false negative, which we did not know. Once the crossmatch during the procedure revealed the positive antibody screen and the blood bank informed us about the multiple hour-long delays in receiving the correct blood, we had to make the difficult decision to hold on the surgery or proceed with possible transfusion requirement and the consecutive risk of acute or delayed hemolytic transfusion reactions. To prevent such scenarios, it is crucial to implement a life-long medical alert in the hospital’s EMR report system for patients with at least one positive type and screening result. If the former antibody screening would have been known in our case, we could have prevented such a scenario. Friedberg’s study showed that 9% of type and screens are not finished before surgery starts and carry the risk of discontinuation or delay of the surgery once a positive result is shown [2]. If an alert similar to an allergy alert in the EMR system would exist, a positive antigen screen surprise would be prevented. After this incident, our hospital introduced such an alert in our electronic medical report system for patients with positive antibody screening.

Uncross-matched type O negative blood may be given to patients with positive antibody screens who experience emergent intraoperative hemorrhage. The risk-benefit ratio of acute hemorrhage versus acute or delayed hemolytic transfusion reactions must be carefully assessed. If appropriate units are unavailable, then the benefit of the blood transfusion must be balanced against the risk of alloimmunization, and a restrictive transfusion policy should be employed. The smaller the amount of the patient’s native blood that remains in circulation, the lower will be the risk of a transfusion reaction occurring [3]. Garratty and Petz recommend considering uncross-matched type O negative blood for transfusion in patients with positive screening tests and life-threatening anemia [8].

Although very rare, alloimmunization with anti-U antibodies can cause hemolytic transfusion reactions [9]. Only 24 cases of anti-U immunization have been reported in the literature. All reported cases were pregnant women from Sub-Saharan Africa’s ethnic groups [10]. The pathophysiology of anti-U antibody, similar to Rh isoimmunization, follows pregnancy or prior blood transfusion (in 1.2% of African descent). The U antigen occurs almost universally in the Caucasian population, causing a limited availability of cross-matched blood suitable for transfusion in patients with anti-U antibodies [11].

## Conclusions

Perioperative RBC transfusions are common. RBC transfusion requires prior antigen screening. False-negative results can lead to acute and delayed hemolytic transfusion reactions, leading to increased morbidity and mortality. This case report emphasizes the importance of life-long alerts in hospital EMR systems for patients with positive antigen screening.

## References

[1] Frank SM, Savage WJ, Rothschild JA, Rivers RJ, Ness PM, Paul SL, Ulatowski JA (2012). Variability in blood and blood component utilization as assessed by an anesthesia information management system. Anesthesiology.

[2] Friedberg RC, Jones BA, Walsh MK (2003). Type and screen completion for scheduled surgical procedures. A College of American Pathologists Q-Probes study of 8941 type and screen tests in 108 institutions. Arch Pathol Lab Med.

[3] Murphy M, BenAvram D (2019). American Association of Blood Banks. Recommendations on the use of group O red blood cells. https://www.aabb.org/docs/default-source/default-document-library/resources/association-bulletins/ab19-02.pdf.

[4] Goodnough LT (2012). Operational, quality, and risk management in the transfusion service: lessons learned. Transfus Med Rev.

[5] Theis SR, Hashmi MF (2022). Coombs Test. https://www.ncbi.nlm.nih.gov/books/NBK547707/.

[6] Goldman L, Schafer A (2012). Goldman’s Cecil Medicine. 24th edition.

[7] Buchta C, Coucke W, Mayr WR, Müller MM, Körmöczi GF (2020). To win the battle, first know your enemy: error rates in immunohematology external quality assessment results. Transfus Med Hemother.

[8] Garratty G, Petz LD (1993). Transfusing patients with autoimmune haemolytic anaemia. Lancet.

[9] Rothman IK, Alter HJ, Strewler GJ (1976). Delayed overt hemolytic transfusion reaction due to anti-U antibody. Transfusion.

[10] Ringressi A, Biagioni S, Mello G, Graziani G, Mecacci F (2012). Anti-U alloimmunisation in a pregnant woman from Niger. Blood Transfus.

[11] Wiener AS, Unger LJ, Cohen L (1954). Distribution and heredity of blood factor U. Science.

